# Harnessing the immune system in glioblastoma

**DOI:** 10.1038/s41416-018-0258-8

**Published:** 2018-11-05

**Authors:** Nicholas F. Brown, Thomas J. Carter, Diego Ottaviani, Paul Mulholland

**Affiliations:** 1 Department of Oncology, University College London Hospitals, 250 Euston Road, London, NW1 2PG UK; 2 Barts Cancer Institute, Barts and the London School of Medicine and Dentistry, Queen Mary University of London, London, EC1M 6BQ UK; 3 UCL Cancer Institute, University College London, 72 Huntley Street, London, WC1E 6DD UK

## Abstract

Glioblastoma is the most common primary malignant brain tumour. Survival is poor and improved treatment options are urgently needed. Although immunotherapies have emerged as effective treatments for a number of cancers, translation of these through to brain tumours is a distinct challenge, particularly due to the blood–brain barrier and the unique immune tumour microenvironment afforded by CNS-specific cells. This review discusses the immune system within the CNS, mechanisms of immune escape employed by glioblastoma, and the immunological effects of conventional glioblastoma treatments. Novel therapies for glioblastoma that harness the immune system and their current clinical progress are outlined, including cancer vaccines, T-cell therapies and immune checkpoint modulators.

## Introduction

Glioblastoma is the most common malignant primary brain tumour in adults.^1^ Survival is poor, with a median survival of 14.6 months with standard treatment of surgical debulking followed by external-beam radiotherapy with concurrent temozolomide (TMZ) chemotherapy and adjuvant TMZ chemotherapy.^2^ In the last decade, clinical trials investigating targeted therapies have failed to demonstrate any significant improvement in survival, and innovative new treatments are urgently needed.^3^ Recently, focus has shifted towards novel strategies that modulate the immune response towards the tumour and the surrounding tumour microenvironment.

Recognition and eradication of malignant cells via immune surveillance of tumour-associated antigens (TAAs) is a key function of the immune system.^4^ TAAs typically represent peptides which are present within tumour cells but usually absent in the surrounding normal tissue. In glioblastoma, these antigens most commonly fall into three main classes; (i) aberrantly expressed non-mutated self-antigens, (ii) mutated self-antigens and (iii) unique antigens or neo-antigens - novel peptide sequences which are the result of somatic mutations in the cancer genome.^5^ Tumours manipulate the immune system to avoid detection of TAAs and to facilitate their own growth and survival.^6^

Immunotherapy aims to harness the immune system against tumours, with breakthroughs observed in a number of cancers, most notably malignant melanoma and haematological malignancies. However, translating these approaches into therapies for primary brain tumours represents a distinct challenge due the unique tumour microenvironment and distinctive immune system within the CNS.

The CNS was traditionally considered immune privileged due to (i) the presence of the blood–brain barrier (BBB) that restricts access to immune cells, (ii) an absence of conventional lymphatic drainage restricting the trafficking of antigens to lymph nodes,^7^ (iii) a scarcity of specialised antigen-presenting cells,^8^ and iv) downregulation of the major histocompatibility complex (MHC) expression in normal brain parenchyma, limiting antigen presentation.^9^ In recent years, this dogma has been eroded with substantial evidence now demonstrating that these interlinked factors tightly regulate a fully functional, innate and adaptive immune system within the CNS (Table 1).

**Table 1:** Current understanding of the CNS immune system

Characteristic	Current understanding
Blood–brain barrier	Leukocyte entry into the CNS is mediated by adhesion signals on endothelial cells (ECs) of the BBB. Limited expression of adhesion signals on ECs in healthy CNS results in low immune surveillance.^157^ In disease processes, infiltration of specific immune cell subsets is observed, which may be driven by BBB ECs or by immune cells within the CNS.^158^ While naive T cell are absent within the CNS, activated T cells cross the BBB as patrolling memory T cells and regulatory T-cells; preventing inappropriate inflammation^8^ and facilitating myelin regeneration.^159^
Lymphatic drainage	Extracellular fluid in the CNS is composed of cerebrospinal fluid (CSF) and interstitial fluid (ISF). CSF is mainly contained within the ventricular system and subarachnoid space, and drains directly into deep cervical and lumbar lymph nodes via lymphatic vessels associated with the nasal mucosa, dura mater and nerve roots.^7, 160^ ISF is found in the extracellular spaces of CNS parenchyma and drains into cervical lymph nodes via intramural perivascular drainage pathways in cerebral artery walls.^7, 160^ Both CSF and ISF may communicate within the brain parenchyma via the glial lymphatic system, a perivascular channel system formed by astroglial cells which removes waste proteins and macromolecules.^161, 162^ Tissue metabolites found within the glial lymphatic system traffic to deep cervical and lumbar lymph nodes via CSF. Within these lymph nodes, T cells may become primed and activated to recognise CNS-specific antigens.^7, 156^
Antigen-presenting cells	Three subsets of dural macrophage populations have been identified, named for their location in the CNS.^163^ Meningeal macrophages and choroid plexus macrophages are bone marrow derived, while perivascular macrophages appear to originate from haematopoietic stem cells in the embryonic yolk, an origin they share with microglial cells.^7^ These macrophage populations can all act as antigen-presenting cells. The position of perivascular macrophages allows them to sample both blood and CNS ISF, implying a possible role in communication between the CNS and periphery.^163^ As well as this, there is evidence from in vivo models that brain parenchyma is completely screened every few hours by resting microglia.^164^
Antigen presentation via MHC expression	CNS antigen presentation is thought to occur at the BBB by microglia, dural macrophages or dendritic cells at so-called CNS ‘immune gateways’ where MHC is expressed.^156^ These gateways also act as entry ports for activated T cells, influx of which can be followed by monocyte recruitment that amplifies inflammatory reactions within CNS.^157^

In this review, we describe the features of the immune system in the CNS, discuss the immune evasion strategies of glioblastoma, the molecular properties of its microenvironment, and the current clinical evidence supporting a role for immunomodulation in the treatment of glioblastoma.

## Glioblastoma tumour microenvironment and mechanisms of immune escape

**Fig. 1:**
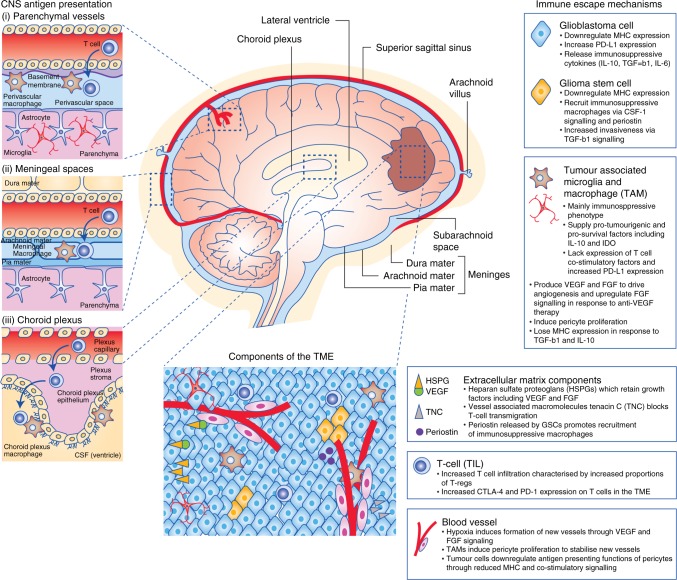
Immune gateways (left). In addition to the resident microglia, there are three distinct macrophage populations within the CNS present at so-called ‘immune gateways’ that act as ports of entry for activated T cells into the CNS. Perivascular macrophages, derived from the embryonic yolk sac, are located around parenchymal vessels (top). The other two populations, derived from bone marrow, are located in the meningeal spaces (middle) and the choroid plexus (bottom) (adapted from ref. ^156^). Immune evasion in glioblastoma (right): the immunosuppressive tumour microenvironment (TME) of glioblastoma is the result of complex interactions between tumour cells, microglia, tumour-associated macrophages (TAMs), components of the extracellular matrix and tumour infiltrating lymphocytes (TILs), which are predominantly regulatory in phenotype (T-regs). Hypoxia promotes angiogenesis of abnormal blood vessels, further driving tumour growth (adapted from ref. ^10^)

Glioblastoma arises from glial cells, with surrounding brain parenchyma comprising CNS-specific cells including astrocytes, neurons and microglia, and a distinctive extracellular matrix (ECM) composition.^10^ Glioblastoma induces a TME characterised by immunosuppressive cytokines secreted by tumour cells, microglia and tumour-associated macrophages (TAMs). These factors, notably interleukin-6, interleukin-10, transforming growth factor-beta (TGF-β), and prostaglandin-E collectively inhibit both the innate and adaptive immune systems with suppression of NK activity and T-cell activation and proliferation, induction of T-cell apoptosis, downregulation of MHC expression, and skew of TAMs towards an M2 (immunosuppressive) phenotype.^11^ The TME is also characterised by tissue hypoxia provided by irregular vascularity and high-tumour oxygen consumption. Tissue hypoxia activates the immunosuppressive STAT3 pathway, leading to hypoxia-inducible factor-1 alpha (HIF-1α) synthesis, activation of regulatory T cells (T-regs) and production of vascular endothelial growth factor (VEGF),^11^ which can inhibit the maturation and function of dendritic cells. These mechanisms of immune escape are discussed below in more detail and summarised in Fig. 1.

### Extracellular matrix composition

ECM proteins commonly found in abundance in peripheral tissues including collagens, laminins and fibronectin are typically only associated with vascular basement membranes within the CNS. Instead, predominant ECM proteins in the glioma TME include glycoproteins, hyaluronic acid and heparan sulphate proteoglycans (HSPGs), which may be concentrated in cancer stem cell niches.^12^ HSPGs, in particular, are upregulated in glioblastoma^13^ and cause retention of heparin-binding angiogenic growth factors such as fibroblast growth factor (FGF) and VEGF, the local release of which promotes tumour angiogenesis and progression.^14^ Furthermore, glioma vasculature can upregulate the vessel-associated macromolecules periostin and tenascin C (TNC), which can promote tumour survival.^15^ These macromolecules can also promote immune evasion with TNC shown to block T-cell movement across glioma-associated blood vessels, preventing their migration into brain parenchyma.^16^ Periostin, when secreted by glioma stem cells, is able to promote recruitment of tumour promoting macrophages from peripheral circulation.^17^

### Macrophages and microglia

Tumour-associated macrophages (TAMs), along with the resident CNS microglia, can constitute up to 30% of the tumour mass.^10^ Transcriptome analysis of TAMs has found that they may possess markers consistent with both M1 (classically activated or immunopermissive) and M2 (alternatively activated or immunosuppressive) phenotypes, incongruous with the traditional M1/M2 dichotomy.^18^ TAM populations can be described both functionally and spatially. For example, CNS-resident microglia exist within the TME alongside distinct populations of bone marrow-derived macrophages (outlined in Table 1),^19^ and recent research has suggested that within the TME, bone marrow-derived macrophages may localise preferentially to the perivascular niche, while resident microglia localise to peritumoural regions.^20^ Accumulation of TAMs expressing CD163 (haemoglobin scavenger receptor) and CD204 (macrophage scavenger receptor), considered ‘M2′ phenotype markers, increases as tumour grade increases.^21^ In glioblastoma, higher CD163 TAM expression correlates with poorer outcomes.^11^ Cancer stem cells in glioblastoma are able to recruit TAMs by overexpression of the macrophage/microglia cytokine colony-stimulating factor-1 (CSF-1).^22^ This induces a pro-tumourigenic microenvironment via release of immunosuppressive factors such as IL-10 and overexpression of the indoleamine 2,3-dioxygenase (IDO) enzyme.^17,23^ M2-TAMs also lack expression of key T-cell co-stimulation molecules^24^ and drive both tumour angiogenesis and resistance to anti-VEGF agents. Angiogenesis is driven through production of pro-angiogenic molecules including VEGF and FGF, with resistance to anti-angiogenics due to upregulation of alternative angiogenic pathways and stimulation of pericytes to proliferate in the perivascular niche. Pericytes are perivascular cells responsible for modulating blood flow, vessel permeability and remodelling, and their proliferation stabilises new vessels.^25,26^ TAMs can also enhance the invasiveness of glioma stem cells (GSCs) via the TGF-β1 signalling pathway.^27^ Further evidence, incongruous with the M1/M2 dichotomy, is that blockade of CSF-1R on the macrophage surface does not deplete glioblastoma TAMs in vivo, but leads to reprogramming of TAMs away from immunosuppressive phenotypes.^28^ Other emerging evidence includes the discovery of a link between tissue hypoxia and macrophage polarisation; with M1 macrophages present within normoxic tumour regions and M2 macrophages present in areas of hypoxia.^29^ These findings support the view that macrophage polarisation is not simply location dependent but instead dependent on distinct signals present in the local microenvironment.^30^ TAMs retain both plasticity and the ability to undergo reprogramming;^31^ characteristics which are potentially exploitable for therapeutic benefit.^32^

### Downregulation of MHC

An effective T-cell immune response requires antigen presentation and subsequent recognition, which is dependent on the co-expression of the human MHC proteins. Comparative analyses of gene expression profiles suggest that invading glioblastoma cells are able to escape immune recognition by downregulating expression of MHC molecules.^33^ The role of antigen presentation within the CNS is thought to fall primarily to the resident microglia, with supporting evidence in vivo demonstrating that microglia are able to cross-present tumour antigens to CD8+ T cells via MHC Class I.^34^ However, the presence of immunosuppressive cytokines such as IL-10 and TGF-ß within the glioblastoma TME cause microglia to lose MHC expression.^11,35^ Pericytes may also play a role in antigen presentation within the CNS, and pericyte MHC Class II expression is shown to increase in response to inflammatory cytokine release.^36^ In the TME, pericytes in contact with glioblastoma cells possess immunosuppressive functions evidenced by changes including a reduction in MHC expression and T-cell co-stimulatory signals.^37^ Low levels of MHC Class I molecules are also found on glioblastoma cancer stem cells, rendering them resistant to T-cell-mediated killing, and thus contributing towards tumour initiation, progression and resistance to therapy.^38^

### T lymphocytes

In patients with glioblastoma, increased T-cell infiltration is found in both tumour (tumour-infiltrating lymphocyte; TIL) and brain parenchyma.^39^ Studies of human tumour samples have shown that this influx is counteracted with a number of events that evade the immune response including further downregulating MHC to prevent antigen presentation,^40^ increasing expression of the inhibitory protein programmed death ligand-1 (PD-L1),^41^ and increased recruitment of immunosuppressive regulatory T cells (T-regs) which express co-inhibitory molecules including cytotoxic T lymphocyte-associated protein 4 (CTLA-4) and programmed death receptor 1 (PD-1).^42,43^ Fourfold more T-regs are found in human glioblastoma samples than benign pituitary adenomas and meningiomas, with CTLA-4 expression on these T-regs threefold that on T-regs in peripheral blood.^42^ PD-L1 expression on circulating monocytes is significantly higher in patients with glioblastoma compared to healthy controls, while expression in tumour infiltrating monocytes is, on average, twice that of circulating monocytes from the same patient.^41^

## Immunological effects of standard therapy

Standard therapy for patients diagnosed with glioblastoma consists of maximal safe debulking surgery, followed by radiotherapy and temozolomide chemotherapy (RT-TMZ).^2^ In addition, high doses of glucocorticoids are frequently administered to reduce tumour-associated and radiotherapy-induced cerebral oedema. These therapies may potentially both augment or diminish immune responses.

Radiotherapy, chemotherapy and glucocorticoid therapy are independently immunosuppressive. Approximately 70% of patients experience clinically significant reductions in their circulating CD4+ lymphocyte counts following standard treatment, persisting for up to a year and associated with early tumour progression.^44^ TMZ-induced lymphopenia is considered a significant limitation to clinical translation of immunotherapies.^44^ Radiation induces M2 macrophages and activation of TGF-β.^45–47^ RT-TMZ has been shown to increase PD-L1 expression in preclinical glioblastoma models, and acquired resistance to radiotherapy has been overcome by PD-L1 blockade in cancer models.^48,49^ Further, as well as depleting systemic lymphocyte counts, RT-TMZ also tilts the balance between regulatory and effector peripheral blood lymphocytes towards an immunosuppressive state.^50,51^ Corticosteroids dampen inflammatory cytokines, deplete T and B lymphocytes, reduce the effectiveness of anti-tumour therapy in rodent models, and their use is associated with poorer survival in patients with glioblastoma.^52–54^

Conversely, standard treatments have been shown to induce immune responses through favourable modulation of the TME and ‘immunogenic cell death’. In addition to cancer cell death through DNA damage and free radical generation, radiation activates an interconnected chain of processes in the TME including inflammation, hypoxia, revascularisation, ECM remodelling and fibrosis.^55^ Radiation induces pro-inflammatory cytokines such as TNF-α, IL-1β, IFN-γ,CXCL9, CXCL10 and CXCL16 which recruit T cells.^56–58^ Sublethal doses of radiation promotes phenotypic changes in tumour cells that promote T-cell recognition and killing, including increasing expression of MHC Class I, co-stimulatory receptors, death receptors and heat-shock proteins.^56,59–63^ Further, evidence suggests RT is able to modulate the tumour vasculature and increase BBB permeability, leading to increased TIL trafficking.^64^ Abscopal responses (tumour response distant from the radiation field), while unusual, illustrate the potential for radiation-induced anti-tumour immunity.^56,65^ TMZ chemotherapy induces hypermutation of the tumour, and increased mutational load correlates with response to immune checkpoint inhibitors in a number of cancers including melanoma, NSCLC and urothelial cancers.^66–71^ Durable responses to nivolumab were observed in a case report of two patients with high mutational burden glioblastoma resultant from biallelic mismatch repair deficiency.^72^ In murine glioma models, TMZ has been found to increase priming of tumour antigen-specific CD4 and CD8 T cells.^73^ While associated with early progression, in one study of patients with glioblastoma, lymphopenia following TMZ was associated with better survival.^74^ Clinical evidence of immune-stimulation has been observed in vaccine trials of a dendritic cell vaccination where pre-treatment with RT-TMZ increased tumour antigen-specific T cells.^75^

This delicate balance between stimulation or suppression of immune responses by standard therapies is still being elucidated. Potentially, careful timing to permit lymphocyte recovery, dose modification or local rather than systemic administration of standard therapies may permit immune priming of tumours without negation due to immunosuppressive sequelae.^63,76^ Recently, adding tumour-treating fields (alternating electric fields delivered via a transducer array applied to the scalp) to standard therapy has been shown to prolong survival.^77^ Whether this may enhance immune responses is unknown and further clinical trials are underway (Table 2).^78^

**Table 2:** Examples of ongoing clinical trials of immunotherapies in glioblastoma. Identifier from www.clinicaltrials.gov

Target	Therapy	Setting	Phase	Identifier
*Checkpoint inhibitors*				
PD-1	Radiotherapy + temozolomide + either nivolumab or placebo	Newly diagnosed	II	NCT02667587
	Radiotherapy + either nivolumab or temozolomide	Newly diagnosed	III	NCT02617589
	Radiotherapy + temozolomide + pembrolizumab	Newly diagnosed	I/II	NCT02530502
	Nivolumb + either high or low dose bevacizumab	Relapsed	II	NCT03452579
	Pembrolizumab ± bevacizumab	Relapsed	II	NCT02337491
	Pembrolizumab + surgery	Relapsed	II	NCT02337686
PD-1 + CTLA-4	Ipilimumab and/or nivolumab in combination with temozolomide	Newly diagnosed	I	NCT02311920
	Nivolumab vs. bevacizumab; Nivolumab ± ipilimumab	Relapsed	III	NCT02017717
	Tumour-treating fields + nivolumab ± ipilimumab	Relapsed	II	NCT03430791
4-1BB + LAG-3 + PD-1	Anti-LAG-3 or urelumab ± nivolumab	Relapsed	I	NCT02658981
*Vaccines*				
Dendritic cell vaccine	DCVax-L or placebo with radiotherapy + temozolomide	Newly diagnosed	III	NCT00045968
	ICT-107 or placebo with radiotherapy + temozolomide	Newly diagnosed	III	NCT02546102
	ADCTA-G + radiotherapy + temozolomide	Newly diagnosed	II	NCT02772094
	ICT-121	Relapsed	I	NCT02049489
Peptide vaccine	Temozolomide + IMA950 + Poly-ICLC (after radiotherapy)	Newly diagnosed	I/II	NCT01920191
	Bevacizumab ± DSP-7888	Relapsed	II	NCT03149003
	Temozolomide + APVAC + Poly-ICLC + GM-CSF (after radiotherapy)	Newly diagnosed	I	NCT02149225
	Mutation-derived tumour antigen vaccine + Tumour-treating fields + temozolomide	Newly diagnosed	I	NCT03223103
*Adoptive cell therapies*				
T cell	CMV-specific cytotoxic T cells + temozolomide ± surgery	Relapsed	I/II	NCT02661282
T cell/CAR-T	Anti-Her2 CAR CMV-specific T cells	Relapsed	I	NCT01109095
CAR-T	Anti-IL13Rα2 CAR-T cells	Relapsed	I	NCT02208362
CAR-T	Intracerebral Anti-EGFRvIII CAR-T	Relapsed	I	NCT03283631
CAR-T	Intracranial Anti-Her2 CAR-T Cells	Relapsed	I	NCT0242297
CAR-T	Anti-EGFRvIII CAR-T cells	Relapsed	Pilot	NCT02209376
*Viral therapy*				
Adenovirus	Delta-24-RGD adenovirus	Recurrent	I/II	NCT01582516
Adenovirus	DNX-2401 + temozolomide	Recurrent	I	NCT01956734
Adenovirus	DNX-2401 + pembrolizumab	Recurrent	II	NCT02798406

## Immunotherapeutic approaches and clinical progress in glioblastoma

**Fig. 2:**
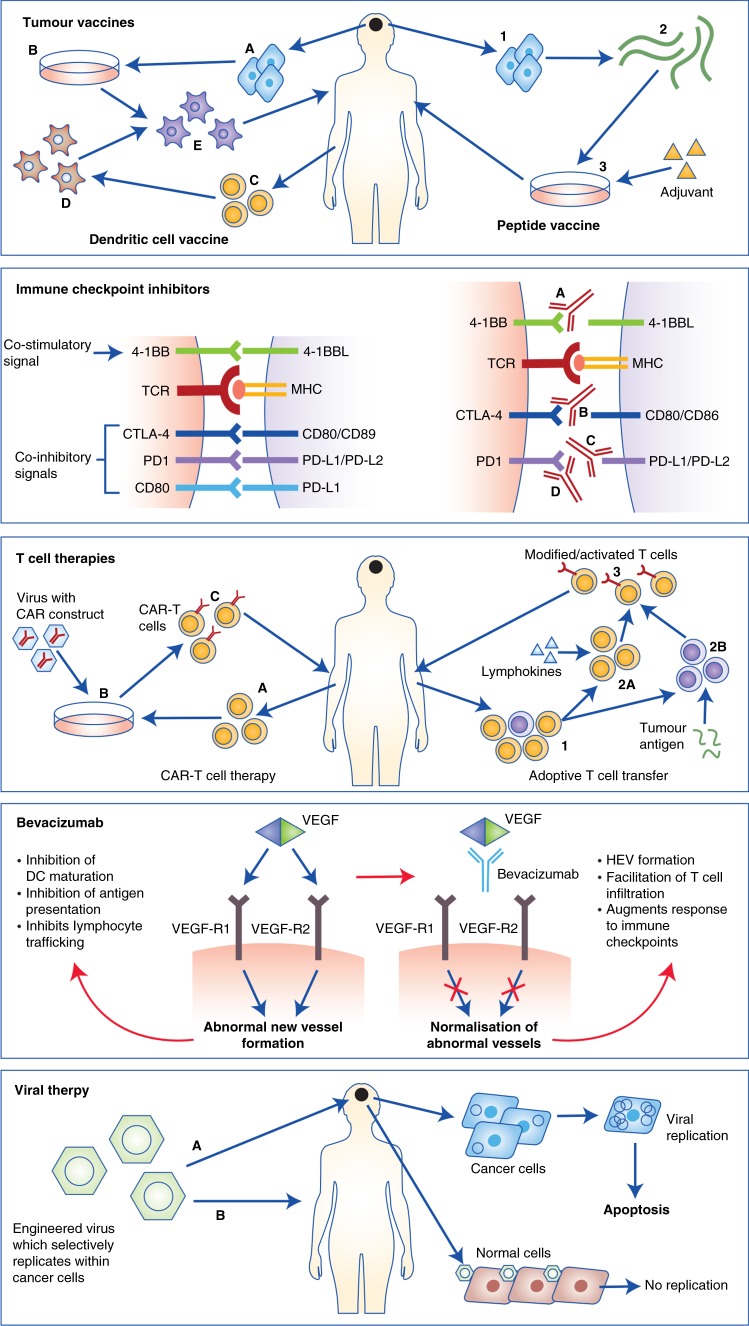
Immunotherapeutic approaches in glioblastoma. From top: Tumour vaccines: There are two main approaches; In dendritic cell vaccination (left) tumour cells are isolated at surgery (**a**), and processed to form a tumour lysate (**b**). Apheresis is done to isolate immature monocytes (**c**), which are then activated ex vivo into immature dendritic cells (**d**). Finally, these dendritic cells are matured and activated using tumour lysate and then returned to patients as intra-dermal injection (**e**). In peptide vaccination (right) tumour cells are isolated after surgery (1), and then further processed to isolate tumour antigens (2). These are then artificially produced and processed into a HLA-matched vaccine (3), which is then returned to the patient as an intradermal injection. Immune Checkpoint Inhibitors: While T-cell responses are initiated through the interaction of MHC Class I/II bound antigen with the T-cell receptor (TCR), the amplitude and quality of this response is regulated by a balance of co-inhibitory and co-stimulatory signals; commonly referred to as immune checkpoints. Checkpoint inhibitors function to either mimic co-stimulatory signals or prevent co-inhibitory signals. Therapies targeting a number of checkpoints are in development, including 4-1BB (**a**), CTLA-4 (**b**), PD-L1 (**c**) and PD-1 (**d**). T-cell therapies: In CAR-T cell therapy (left), autologous T cells are isolated and expanded (**a**) and the CAR construct inserted with viral vectors (**b**). Autologous CAR-T cells are then returned to the patient as an infusion (**c**). In adoptive cell transfer (right), following T-cell isolation and expansion (1), T cells are either activated ex vivo using lymphokines (2A) or selected for a specific tumour antigen (2B). Cells are then expanded and returned to patients as an infusion (3). Bevacizumab: Right: Unopposed VEGF signalling within tumours induces new blood vessel formation, inhibitors dendritic cell (DC) maturation, antigen presentation and T-cell trafficking. Left: In the presence of bevacizumab, VEGF signalling is blocked resulting in vessel normalisation, formation of high endothelial venules (HEVs) and facilitation of T-cell trafficking, augmenting response to immune checkpoint inhibitors. Oncolytic viral therapies: Oncolytic viruses are engineered to replicate preferentially in glioblastoma cells (due to lack of tumour suppressor function). Viruses are delivered either directly into tumours (**a**) or intravenously (**b**) if able to travel across the BBB. Within normal cells, viruses do not replicate due to intact tumour suppressor apparatus. However, within tumour cells, viruses replicate and induce apoptosis

Early trials in glioblastoma largely administered systemic or intra-tumoural cytokines, with development limited by significant toxicity with only modest clinical benefit.^79,80^ Arguably the most promising of these, IFN-γ, possesses anti-proliferative, anti-angiogenic and pro-apoptotic functions and is able to increase antigen presentation through upregulation of MHC Class I and II, as well as mediating immune cell infiltration.^81^ However, IFN-γ also induces PD-L1 expression as well as promoting T-reg development. It is also possible that glioblastoma cells exploit IFN- γ through low level upregulation to induce PD-L1 expression and escape immune cell detection.^82^ These factors may help to explain why these early immunotherapeutic approaches were disappointing,^83^ and why current focus has moved towards therapies that either prime immune cells to target-specific tumour-associated antigens, or modulate the TME to reverse immune escape (Fig. 2). As yet, only two phase III trials have completed: the EGFRvIII-targeted vaccine rindopepimut in newly diagnosed glioblastoma; and the checkpoint inhibitor nivolumab in relapsed glioblastoma; with neither demonstrating survival advantage.^84,85^ Phase III trials of checkpoint inhibitors and dendritic cell vaccines are ongoing, with a large number of agents in earlier stages of clinical development including peptide vaccines, adoptive T-cell therapies, oncolytic viruses (OV) and combination therapies (Table 2).

### Immune checkpoint inhibitors

The amplitude and quality of T-cell responses are regulated by a balance of co-inhibitory and co-stimulatory signals, termed immune checkpoints.^66^ Tumours exploit these safety mechanisms to render T cells inactive within the TME. In glioma, higher expression of PD-L1 expression correlates with increasing tumour grade and is associated with poor survival in glioblastoma. A total of 88–100% of glioblastomas express PD-L1, and it is also expressed on microglia and TAMs within the TME.^11,86–89^ Checkpoint inhibitors are monoclonal antibodies that either inhibit the activity of immune checkpoints, or mimic ligand binding. Checkpoint inhibitors targeting PD-1 (nivolumab/pembrolizumab), PD-L1 (atezolizumab/durvalumab/avelumab) and CTLA-4 (ipilimumab) are licensed for use in various cancers, with pembrolizumab licensed by the FDA for mismatch repair deficient and microsatellite instability-high tumours regardless of the tissue of origin. Although overall nivolumab was overall found not to improve survival in patients with relapsed glioblastoma, a small subset of patients did have a durable response, and analysis of their tumour biomarkers and immune responses may guide future trials.^84^ Initially within this trial, an exploratory cohort of patients were treated with nivolumab and ipilimumab (licensed for use in melanoma), but this combination was less well tolerated compared to nivolumab monotherapy and so was not included in the main trial.^90^ Nivolumab is currently under investigation in combination with radiation ± TMZ in newly diagnosed patients, and trials of other checkpoint inhibitors are ongoing or awaiting final results (Table 2). Early interim reports from phase II trials of pembrolizumab in relapsed glioblastoma and durvalumab with bevacizumab in newly diagnosed/relapsed glioblastoma are encouraging.^91,92^ Checkpoint inhibitors are associated with a variety of immune-mediated toxicities.^93^ Notably for glioblastoma, transient increases in tumour size can occur due to inflammatory infiltrates, which within the fixed size of the cranial vault may cause raised intracranial pressure, requiring urgent medical or surgical intervention.^94^

### Tumour vaccines

Cancer vaccination is achieved in several ways (Fig. 2). The first involves administering a tumour-specific antigen (or combination of antigens), which can then be trafficked to antigen-presenting cells for presentation to T cells to elicit an immune response. Drawbacks are that it is HLA subtype specific, and that it relies upon expression of the specific antigen within the patient’s tumour.^95^ The second method is to collect autologous dendritic cells, prime them ex vivo with the patient’s tumour antigens, and then administer them back into the patient intradermally; a process referred to as dendritic cell vaccination.^39^ The only vaccine therapy to have completed evaluation is rindopepimut, an EGFRvIII-targeted vaccine, which did not improve survival when administered alongside standard therapy in patients with newly diagnosed EGFRvIII mutant glioblastoma.^85^ However, an exploratory outcome found improved survival in patients with significant residual disease. Various vaccines are in earlier stages of development, and discussed more fully elsewhere, with several promising candidates.^96^ HSPPC-96, an autologous vaccine derived from antigenic tumour peptides bound to heat-shock protein, reported a promising 6 month survival rate of 90% in a phase II trial in 41 patients with recurrent glioblastoma.^97^ AFTV is an autologous vaccine derived from formalin-fixed tumour samples which reported a favourable 3 year survival rates of 38% in a phase I/IIa trial in 24 patients with newly diagnosed glioblastoma treated with AFTV in addition to standard therapy.^98^ A peptide vaccine that selected 4 of 14 pre-selected antigens based upon pre-existing antigen-specific IgG responses has demonstrated the feasibility of personalised vaccines in a phase I trial in 12 patients with recurrent glioblastoma.^99^

### T-cell therapies

Chimeric antigen receptor T-cell (CAR-T) therapy is an adoptive cell therapy in which autologous T cells are isolated, genetically modified to express chimeric receptors targeting tumour antigens, and re-administered to the patient subsequent to lymphodepletive chemotherapy. This strategy bypasses the need for MHC-dependent co-stimulation (Fig. 2) due the fusion of antigen-binding domain to T-cell activation and co-stimulatory domains.^100^ Impressive clinical responses in patients with treatment-refractory lymphoma treated with CD19-targeted CAR-T cells have led to the recent licensing of the first approved CAR therapies.^101,102^ However, deaths from cytokine release storms have occurred due to the potency of the immune response generated.^103^ In contrast to B cell aplasia which is tolerable, in solid tumours on-target off-tumour toxicity may cause critical organ dysfunction, which limits use to antigens that are highly tumour specific.^103,104^ In addition, the immunosuppressive TME in solid tumours reduces CAR-T trafficking to the tumour, and subsequent proliferation and persistence.^104^

Early-phase clinical trials in glioblastoma are underway investigating CAR-T therapies targeting IL-13 receptor alpha 2 (IL-13Rα2), epidermal growth factor receptor variant III (EGFRvIII) and human epidermal growth factor receptor 2 (Her-2) (Table 2). IL-13Rα2 is abundantly expressed on the majority of glioblastomas in both differentiated and stem-cell like cells, with limited expression in normal brain.^105^ EGFRvIII is a mutated form of wild-type EGFR that is tumour-specific and present in approximately one quarter of patients with glioblastoma.^106^ Reported frequency of Her-2 expression in glioblastoma varies greatly from 0 to 42%.^107–109^ Early reports highlight striking responses.^110–112^ Other adoptive cell therapy strategies in early clinical development include cytotoxic T lymphocytes specific for antigens such as cytomegalovirus (present in over 90% of glioblastoma tumours, but not surrounding brain), lymphokine-activated cytotoxic T cells and natural killer cells.^113–115^

Current approaches to enhancing CAR-T cell therapy in solid tumours include strategies to incorporate additional intracellular signalling domains that enhance proliferation and persistence (e.g. IL-12-secreting armoured CARs) and provision of user control over T-cell immune activation to control toxicity, for example conditional CARs that require small molecule activation.^116^ CARs have also been inserted into other immune cell subsets (e.g. CAR-NK cells) with promising preclinical reports.^117,118^

### Oncolytic viruses

OVs are native or genetically engineered viruses that promote anti-tumour responses through selective replication within cancers cells resulting in cell lysis or immunogenic cell death.^119,120^ Glioblastoma is susceptible to OVs in preclinical models.^121–123^ Overcoming viral exclusion by the blood–brain barrier has been achieved by direct tumour injection of OVs, or by using viruses known to have good CNS penetration (e.g. parvovirus).^124^ Early phase trials have established OVs as safe in glioblastoma, but as yet efficacy has been modest.^125^ Current strategies are focussing on targeted OVs and combination therapy (Table 2).^126,127^

### Bevacizumab

Bevacizumab is an anti-VEGF monoclonal antibody. It is used as a steroid-sparing agent in glioblastoma for the management of cerebral oedema, although has not demonstrated a survival benefit and is licensed outside of the EU for this indication.^128–130^ As well as modulating vasculature, VEGF inhibits dendritic cell maturation, antigen presentation, and lymphocyte trafficking into tumours.^131,132^ As such, bevacizumab is finding a new role as an adjunct to immunotherapies, possibly via the normalisation of tumour vasculature and induction of high endothelial venule (HEV) formation within tumours, both of which facilitate T-cell infiltration and activation.^133,134^ Anti-VEGF therapy induces transient tumour vessel normalisation, but studies support that lower doses than those routinely used in clinical practice result in a longer ‘normalisation window’.^135^ Combination ipilimumab and bevacizumab in patients with melanoma suggests VEGF inhibition enhances anti-tumour immune responses.^136^ Several combination anti-VEGF/immunotherapy studies are underway in glioblastoma (Table 2), with interim data from a phase II trial of the anti-PD-L1 agent durvalumab in combination with bevacizumab reporting apparent clinical activity in a subset of bevacizumab-naive patients.^74^

## Conclusions and future directions

Cancer treatment is moving towards personalised care with the ability to therapeutically target-specific tumour characteristics. Immunotherapies currently in clinical development will likely benefit a subset of patients with glioblastoma; however, no reliable biomarker currently exists to predict these patients.

Unlike most cancers, glioblastomas rarely metastasise, affording the unusual opportunity of potentially using localised therapy for advanced disease.^137^ Based on observations in melanoma that increased tumour burden correlates with a poorer response to the anti-PD-1 antibody pembrolizumab,^138^ it is possible that surgical debulking in glioblastoma may enhance the effectiveness of immunotherapy by diminishing the immunosuppressive TME. Ongoing trials of nivolumab in newly diagnosed glioblastoma that are stratified by the extent of resection may provide insight. If so, the role of surgery may be extended to recurrent disease (when it is not routinely performed) as an adjunct to immunotherapy.

Various devices are available to bypass the BBB and deliver therapies directly into a tumour or tumour cavity, reducing systemic toxicity, and potentially modulating the TME more favourably than systemic treatment.^139–141^ Intracranial administration via catheter delivery is currently under investigation in clinical trials of CAR-T cell therapy, with case reports of clinical efficacy.^112^

There are also new avenues being explored to augment the efficacy of immunotherapy. Recent evidence shows that distinct gut microbial composition, or microbiome can affect and even enhance responses to checkpoint inhibitors,^142^ and that depletion of intestinal bacteria with antibiotics can diminish the patient’s response to immunotherapy.^143,144^ Future breakthroughs could manipulate the microbiome towards a status that promotes anti-tumour immune responses.^145^ Another growing field is thermal therapy, with evidence that hyperthermia can mediate immunogenic cell death.^146^ It is also critical that the TME if fully characterised to determine how it can be further targeted for cancer immunotherapy. T-cell metabolism is a developing field, and a promising therapeutic target is IDO, a tryptophan catabolic enzyme overexpressed in several tumour types that is thought to create an immunosuppressive microenvironment by inhibiting T-cell immunity.^147,148^

Tumour heterogeneity is well characterised in glioblastoma, and may contribute towards immune cell heterogeneity within tumours.^149,150^ Glioblastoma heterogeneity is at least partly maintained by extracellular vesicles (EVs) that contain molecules including DNA, RNA and proteins. The shedding and uptake of these vesicles from tumour cells form an additional communication network capable of modulating the TME. Recent evidence has also shown that PD-L1 is expressed on glioblastoma EVs.^151,152^ Further, vascular heterogeneity exists, with vessel co-option (glioblastoma migration along existing blood vessels) occurring alongside angiogenesis, increasing the complexity of heterogeneity within glioblastoma.^153^ Further research into these factors, including utility of techniques such as single-cell RNA sequencing to characterise both tumour, and immune cell heterogeneity^154,155^ may facilitate a future shift towards more personalised treatment.

In summary, the CNS has an active immune system and glioblastoma employs a number of strategies to evade immune-mediated death. To design therapies that effectively harness the immune system against glioblastoma, a deeper understanding of the complex interactions between tumours and the tightly regulated immune system in the CNS is needed. Future therapies will need to address multi-dimensional challenges including identifying truly tumour-specific antigens to target, increasing tumour immunogenicity, targeting the immune microenvironment and controlling toxicity of therapy.
